# Effect of Fructooligosaccharides Fraction from *Psacalium decompositum* on Inflammation and Dyslipidemia in Rats with Fructose-Induced Obesity

**DOI:** 10.3390/nu6020591

**Published:** 2014-01-29

**Authors:** Héctor Merino-Aguilar, Daniel Arrieta-Baez, Manuel Jiménez-Estrada, Gil Magos-Guerrero, René Javier Hernández-Bautista, Ana del Carmen Susunaga-Notario, Elizabeth Hernández-Pérez, Norma Edith López-Díazguerrero, Julio Cesar Almanza-Pérez, Gerardo Blancas-Flores, Rubén Román-Ramos, Francisco Javier Alarcón-Aguilar

**Affiliations:** 1 Programa de Doctorado en Ciencias Biológicas y de la Salud. D.C.B.S., Universidad Autónoma Metropolitana-Iztapalapa, Apdo-Postal 55-535, México D.F. C.P. 09340, Mexico; E-Mail: hmerino@ciencias.unam.mx; 2 Instituto Politécnico Nacional-CNMN, Calle Luis Enrique Erro s/n, Unidad Profesional Adolfo López Mateos, Col. Zacatenco, México D.F. C.P. 07738, Mexico; E-Mail: danielarrieta@hotmail.com; 3 Instituto de Química, Universidad Nacional Autónoma de México, Coyoacán, México D.F. C.P. 04510, Mexico; E-Mail: manueljemex@gmail.com; 4 Facultad de Medicina, Universidad Nacional Autónoma de México, Coyoacán, México D.F. C.P. 04510, Mexico; E-Mail: gamagos@unam.mx; 5 Programa de Doctorado en Biología Experimental, D.C.B.S., Universidad Autónoma Metropolitana-Iztapalapa, Apdo-Postal 55-535, México D.F. C.P. 09340, Mexico; E-Mails: rjhb11@yahoo.com.mx (R.J.H.-B.); qfbsus@yahoo.com.mx (A.C.S.-N.); 6 Departamento de Ciencias de la Salud, D.C.B.S., Universidad Autónoma Metropolitana-Iztapalapa, Apdo-Postal 55-535, México D.F. C.P. 09340, Mexico; E-Mails: sila@xanum.uam.mx (E.H.-P.); norm@xanum.uam.mx (N.E.L.-D.); jcap@xanum.uam.mx (J.C.A.-P.); gera@xanum.uam.mx (G.B.-F.); rrr@xanum.uam.mx (R.R.-R.)

**Keywords:** *Psacalium decompositum*, asteraceae, fructose, obesity, dyslipidemia, inflammatory cytokines

## Abstract

*Psacalium decompositum*, commonly known as “Matarique,” is a medicinal plant used in Mexico for diabetes mellitus empirical therapy. Previous studies have shown that the fructooligosaccharides (FOS) present in the roots of this plant exhibit a notable hypoglycemic effect in animal models; this effect might be associated with the attenuation of the inflammatory process and other metabolic disorders. In this study, we examined the effects of FOS fraction administration in a fructose-fed rat model for obesity. Phytochemical chromatographic studies (high performance thin layer chromatography and nuclear magnetic resonance) were performed to verify isolation of FOS. 24 male Wistar rats were maintained for 12 weeks on a diet of 20% HFCS in drinking water and chow. Glucose, cholesterol, triglycerides and liver transaminases levels were measured monthly, after administering FOS fraction intragastrically (150 mg/kg/day for 12 weeks), while the levels of inflammatory cytokines were only quantified at the end of the treatments. Rats treated with FOS fraction decreased body weight, cholesterol, triglycerides, and significantly reduced IL-6, IFN-γ, MCP-1, IL-1β and VEGF levels (*p <* 0.05). These results suggest that *P. decompositum* has anti-inflammatory and hypolipidemic properties that might be used as an alternative treatment for the control of obesity.

## 1. Introduction

*Psacalium decompositum* (A. Gray) H. Rob. and Brettell (Asteraceae) is a shrub distributed in the mountains of central and northwestern Mexico and in the southwest region of the United States. The roots are sold in herbal markets and commonly called “Matarique.” A water decoction of the roots of this or other *Psacalium* species is used for the treatment of diabetes mellitus, pains, rheumatism, hepatic and renal colic, gastrointestinal ailments, neuralgia, ulcer and chill by Tarahumaran, Yaqui and Pima traditional healers in the Mexican state of Sonora [1].

The hypoglycemic properties of this plant have been extensively studied; extracts and fractions are effective in reducing glucose levels in normoglycemic and mildly diabetic mice, as well as in temporally hyperglycemic rabbits, but not in severely diabetic animals [2,3].

Jimenez-Estrada *et al*. [4] have reported that the aqueous fraction contains a carbohydrate-type fructan (inulin), which showed hypoglycemic effect in healthy and alloxan-induced diabetic mice. While the hypoglycemic properties of roots of *P*. *decompositum* have been extensively studied, the potential anti-inflammatory and hypolipidemic properties of the fructooligosaccharides-fraction (FOS-fraction) of *P*. *decompositum* have not yet been investigated.

Fructans are polysaccharides of repeating fructose units with a glucose molecule normally attached at the beginning of the polymer; different fructans are found in plants either separately or mixed and they have two different glycosidic linkages, namely β (2-1) linkages (a common feature in inulin type), β (2-6) (normally found in levans) and graminan, or both β (2-1) and β (2-6) [5,6]. Inulin is a linear polyfructan that is mainly comprised of glycosidic linkages β (2-1), whereas the formation of fructooligosaccharides (FOS) is a combination of short and intermediate inulin chains [7].

FOS are classified as indigestible oligosaccharides because of their resistance to digestion in the small intestine, which can improve immunological responses probably through regulation of gastrointestinal microflora, specifically stimulating development of *Bifidobacteria* [8]. Previous studies have reported that FOS intake can prevent hyperlipidemia in rats [9]; effects of FOS on obesity may be due to inhibited increase in visceral adipose tissue and reduced levels of liver triglycerides and nonesterified fatty acids induced by a high-fat diet. Nevertheless, effects of FOS on obesity not yet been fully described [10].

In 2012, Malaguarnera *et al*. reported that FOS administration with *Bifidobacterium longum* reduces tumor necrosis factor type-alpha, C-reactive protein, serum aspartate aminotransferase levels, homeostasis model of assessment-insulin resistance, serum endotoxin, steatosis, and the pathogenesis of non-alcoholic steatohepatitis activity index in patients [11]. There are as of yet no reports about the effects of other similar types of fibers on the inflammatory cytokines. However, these types of fibers have been associated with other activities. A large number of studies in humans and experimental models have evidenced the efficacy of dietary fiber in regulating body weight, food intake, glucose homeostasis, insulin sensitivity and other cardiovascular disease risk factors such as serum lipid profile, hypertension and systemic inflammatory markers. Dietary fiber consists of edible parts of plants or analogous carbohydrates that resist digestion and absorption in the small intestine with complete or partial fermentation in the large intestine. It is a very complex group of substances that includes the indigestible non-starch polysaccharides, cellulose and hemicellulose, oligosaccharides, pectins, gums and waxes [12].

Diverse studies concluded that dietary fructose has a direct impact on hepatic lipid metabolism by bypassing the enzyme phosphofructokinase, the regulatory step imposed on glucose. This allows unregulated flow of fructose-derived carbons into lipogenesis, decreasing lipolysis and increasing plasma fasting and postprandial very low density lipoprotein triacylglycerols, and whole-body lipid oxidation [13].

Patients with obesity suffer from the chronic activation of certain cells in the immune system (monocytes, macrophages, and neutrophils), as well as adipocytes in fat tissue [14] resulting in an abnormal production on the levels of the next cytokines: tumor necrosis factor type-alpha (TNF-α), interleukins 1 and 6 (IL-1α, β and IL-6), interferon-gamma (IFN-γ), monocyte chemotactic protein-1 (MCP-1), vascular endothelial growth factor (VEGF) and leptin, which have also been implicated in the development of insulin resistance, hyperinsulinemia, hypertension, lipid disorders, type 2 diabetes, cardiovascular diseases, some cancers, osteoarthritis, gallstones and mental health problems [15,16,17]. The combination of the immune response with an appropriate metabolic balance is beneficial for maintaining good health. However, it may become deleterious under conditions of metabolic disorders [18].

Therefore, the modulation of these molecules by *P*. *decompositum* might avoid the development of metabolic disorders and complications, in addition to its beneficial effects on obesity.

The aim of the present study was to determine the anti-obesity, anti-inflammatory, and hypolipidemic effects of FOS fraction of roots of *P*. *decompositum* by measuring metabolic parameters and inflammatory cytokines associated with obesity.

## 2. Experimental Section

### 2.1. Plant Material

Roots of *P*. *decompositum* were acquired from the Sonora Herbal Market in Mexico City. The vegetal material was taxonomically identified by Abigail Aguilar Contreras, M.Sc, the botanic expert of the Mexican Institute of Social Security-Herbarium at Mexico City (Herbarium IMSS-M, Voucher Specimen 11489).

### 2.2. Isolation of FOS-Fraction from the Roots of *P. decompositum*

The active fraction (polar fraction) was prepared with some modifications previously described by Alarcon *et al*. [19]. Plant material (1.980 kg) was ground and extracted successively three times at room temperature with hexane and water (7 L × 24 h each one). Water was subsequently removed from the extract under reduced pressure, which resulted in a residue of 427 g (yield 21.5%).

To this material, 500 mL of methanol were added and after 24 h a precipitate was obtained and then freeze-dried (FOS-fraction, 371 g). The FOS-fraction was analyzed by high performance thin layer chromatography (HPTLC, chloroform-*n*-butanol-methanol-acetic acid-water 5.5:11.0:5.0:1.5:2.0, v/v) and was subjected to column chromatography with a Sephadex LH20 column (60 g). Fractions were eluted with a mixture of methanol and water (99:1 and 95:5, v/v). A total of 110 fractions of 25 mL were collected (85 and 25 of each system, respectively) and monitored using HPTLC. Plates were sprayed with an alpha-naphthol reagent and heated at 100 °C.

The fractions were then combined on the basis of similar HPTLC profiles. The major material content isolated was located in fractions 89–91, according to their HPTLC profiles, resulting in a white amorphous solid component. The identity of this component present in the 89–91 fractions was established by nuclear magnetic resonance (^1^H and ^13^C) [4].

### 2.3. HPTLC Procedures

All the samples were applied on 0.2 mm nano-silica gel 60 HPTLC plates. All the plates were developed to a distance of 90 mm with chloroform-*n*-butanol-methanol-acetic acid-water 5.5:11.0:5.0:1.5:2.0 (v/v) as mobile phase at room temperature (around 25 °C). The developed plates were sprayed with aniline-diphenylamine-phosphoric acid solution and heated at 130 °C for 10 min or sprayed with alpha-naphthol (10%) solution and heated at 105 °C for 10 min, to make bands colored clearly.

### 2.4. Nuclear Magnetic Resonance (NMR) Analysis of the FOS Fraction

10 mg of the FOS fraction were dissolved in D_2_O and analyzed on a Varian Gemini spectrometer operating at 500 MHz (^1^H) and 125 MHz (^13^C). Chemical shifts were reported in δ units (ppm) and compared with those previously described [4].

FOS fraction: ^1^H NMR (500 MHz, D_2_O) δ 5.48 ppm (*J =* 3.3 Hz), 4.26 (1H, d, *J =* 8.2) and at 4.12 (1H t, *J =* 8.7, *J =* 8.2), 3.86 (H5, s), 3.76 (H6a, m) and 3.83 (H6b, bs), 3.82 (1H, dg, *J =* 10 Hz), 3.71 ppm (1H, dg, *J =* 10 Hz), 3.56 (1H), 3.84 (1H, m), 3.76 ppm (1H, m). ^13^C NMR (125 MHz, D_2_O) δ 93.23 (C-1), 104.05 (C-2), 81.92 (CH-5), 77.90 (CH-3), 75.20 (CH-4), 62.87 (CH2-6), 61.80 (CH_2_-1).

### 2.5. Animals and Diets

All the experimental protocols were approved by the Local Committees of Ethics on Animal Experimentation (UAM and FM-UNAM). Twenty-four male Wistar rats were obtained from the Laboratory Animal Center of the Metropolitan Autonomous University. The handling of the laboratory animals followed the rules for the care and use of laboratory animals of the Official Mexican Rule (NOM-062-ZOO-1999, revised in 2001) and the International Guide for Caring and Use of Laboratory Animals NRC 2002. Rats at the age of 12 weeks and 200–250 g body weight were maintained in their housing conditions with a controlled humidity (55%) at 21 ± 1 °C temperature with 12/12 h light-dark cycle.

Control diet (2018s Teklad Global 18% protein rodent diet from Harlan Laboratories) contained a percentage of proteins (18.6%), carbohydrates (44.2%) and fat (6.2%). The high-fructose corn syrup (HFCS) diet was 20% in chow and drinking water (Formula 55, v/v dissolved in purified water).

Rats were initially divided into two groups (*n =* 6 for the normal group and *n =* 18 for the fructose (Fru) group), and treated for 12 weeks under the next conditions: the normal group with regular chow and drinking water (negative control), and the second group was kept with 20% fructose in chow and drinking water. After this time, the normal group was kept under the same conditions, while the second group was divided in three groups: the Fru group treated with isotonic saline solution (4 mL/kg/day), the Fru + Beza group treated with bezafibrate (30 mg/kg/day, positive control), and the Fru + FOS group treated with FOS fraction (150 mg/kg/day); all treatments were administered intragastrically and doses were determined based on previous studies of FOS fraction [4]. Groups Fru, Fru + Beza and Fru + FOS were maintained under initial diet conditions and treatments were administrated for 12 weeks. Weight, Lee index—an obesity index used in rodents—was calculated by the cube root of body weight (g) × 10/naso-anal length (mm), for which a value equal to or lower than 0.30 was classified as normal; for values higher than 0.30, the rats were classified as obese, and metabolic parameters were measured every month. Groups were kept in multiple cages being four animals in each.

### 2.6. Oral Glucose Tolerance Test (OGTT)

At the end of experiment, OGTT were performed on 12 h-fasted rats. Glucose was administered into the stomach of the rats through a gastric catheter at the final dose of 2 g/kg body weight (dissolved in purified water), and the glucose levels were measured at 0, 30, 60, 90 and 120 min using an Accutrend Sensor glucometer (Roche). Glycaemia was quantified on days 30, 60 and 90 in fasted animals (12 h) from blood samples drawn from the tail vein. OGTT for non-diabetic rats were performed according to Tai model (the total area under a curve is computed by dividing the area under the curve between two designated values on the X-axis into rectangles and triangles. The total sum of these individual areas thus represents the total area under the curve [20].

### 2.7. Biochemical Parameters

Quantification of total cholesterol (Chol), triglycerides (TG), aspartate aminotransferase (AST or GOT), and alanine aminotransferase (ALT or GPT) was performed with a Reflotron System (Bayer) using blood samples drawn from the tail vein on day 30, 60 and 90 in fasted animals (12 h). Measurements of body weight and circulating levels of cholesterol, triglycerides, glucose and liver transaminases were performed throughout the whole study, while biomarkers of inflammation were only quantified at the end of the treatments.

### 2.8. Cytokines Quantification

At the end of the test (day 90 after treatment), blood samples were obtained from anesthetized animals with pentobarbital by cardiac puncture for cytokine analysis. Serum cytokine levels were quantified using a commercial rat obesity ELISA strip purchased from Signosis, as described in the manufacturer’s protocol in order to get the profile of 8 cytokines (IL-6, IFN-γ, MCP-1, IL-1α, IL-1β, VEGF, TNF-α and leptin). All samples run in single.

### 2.9. Statistical Analysis

Data were expressed as mean ± S.E.M. The significance of the difference between the means of test and control studies was established by an Analysis of Variance (ANOVA) and Tukey’s Multiple Comparison Test. Cytokine analysis was based on percentage of normal. *P* values less than 0.05 were considered to be significant. All statistics were computed using the NCSS 2000 (NCSS software, Kaysville, UT, USA).

## 3. Results

According to Table 1, after 12 weeks, an increasing body weight, Lee index, triglycerides and ALT levels (*p* < 0.05) were observed, while the other parameters were relatively stable compared with the normal group.

**Table 1:** Effect of the FOS-fraction from *P*. *decompositum* on final weight and biochemical parameters in male Wistar rats with 12 weeks fructose feeding.

	Normal	Fru
**Body weight (g)**	307.8 ± 5.6	378.5 ± 10.2 *
**Lee index**	0.28 ± 0.001	0.30 ± 0.002 *
**Glycaemia in fasting (mg/dL)**	101.8 ± 2.5	94.6 ± 4.1
**AUC of glucose tolerance test ** **(** **mg/dL/120 min** **)**	16,800 ± 984	18,000 ± 696
**Chol (mg/dL)**	100 ± 0.0	100 ± 0.0
**TG (mg/dL)**	70 ± 0.00	189 ± 21.8 *
**ALT (UI/L)**	5.08 ± 2.6	19.3 ± 8.0 *
**AST (UI/L)**	10.6 ± 2.8	12.3 ± 8.5

Mean ± SEM (*n =* 6 for Normal group and *n =* 18 for Fru group). * Statistically significant compared with the normal group; (*p* < 0.05). AUC: area under the curve. Normal: normal rats without treatment, FRU: rats treated with high fructose.

Table 2 shows the results obtained from different parameters measured to determine the effect of FOS fraction from *P*. *decompositum*. There was an increase in body weight, cholesterol, triglycerides, ALT, AST and elevated glucose tolerance test glucose at 120 min in animals on a diet of high-fructose compared to animals on a normal diet. The groups treated with bezafibrate and FOS fraction showed differences in body weight, Lee index, cholesterol and triglycerides levels compared with isotonic saline solution-treated animals. They did not show differences in relation to animals on a normal diet. Glycaemia in fasted animals was not altered after the high-fructose diet in any of the experimental groups compared to animals with normal diet, and no reduction was observed in the area under the curve of glucose tolerance of animals treated with bezafibrate and FOS at 120 min. Similar results were observed with ALT and AST.

**Table 2:** Effect of the FOS fraction from *P*. *decompositum* on final weight and biochemical parameters in male Wistar rats with 12 weeks of treatment

	Normal	Fru	Fru + Beza	Fru + FOS
**Final body weight (g)**	490 ± 27.8	610 ± 9.1 *	463 ± 46.0 ^&^	472 ± 15.8 ^&^
**Lee index**	0.295 ± 0.003	0.33 ± 0.000 *	0.303 ± 0.002 ^&^	0.303 ± 0.002 ^&^
**Glycaemia in fasting (mg/dL)**	96 ± 2.4	106.7 ± 2.9	100.7 ± 2.8	99 ± 2.6
**AUC ** **of glucose tolerance test mg/dL/120 min**	16,560 ± 612	19,920 ± 1128 *	18,000 ± 984	18,360 ± 816
**Chol (mg/dL)**	106 ± 4.0	128.5 ± 1.5 *	101.7 ± 1.7 ^&^	102.7 ± 2.7 ^&^
**TG (mg/dL)**	79.5 ± 7.6	205.2 ± 34.4 *	82.5 ± 11.8 ^&^	95.5 ± 19.6 ^&^
**ALT (UI/L)**	3.6 ± 0.8	30.2 ± 3.4 *	18.8 ± 7.9	12 ± 5.0
**AST (UI/L)**	3.2 ± 0.5	38.7 ± 5.1 *	22.7 ± 10.2	15.7 ± 7.3

Mean ± SEM (*n =* 6). * Statistically significant compared with the normal group; ^&^ statistically significant compared to the group treated with Fru (*p* < 0.05); AUC: area under the curve; Normal: normal rats without treatment; FRU: rats treated with high fructose; Fru + Beza: obese rats treated with bezafibrate; Fru + FOS: obese rats treated with FOS.

**Figure 1:**
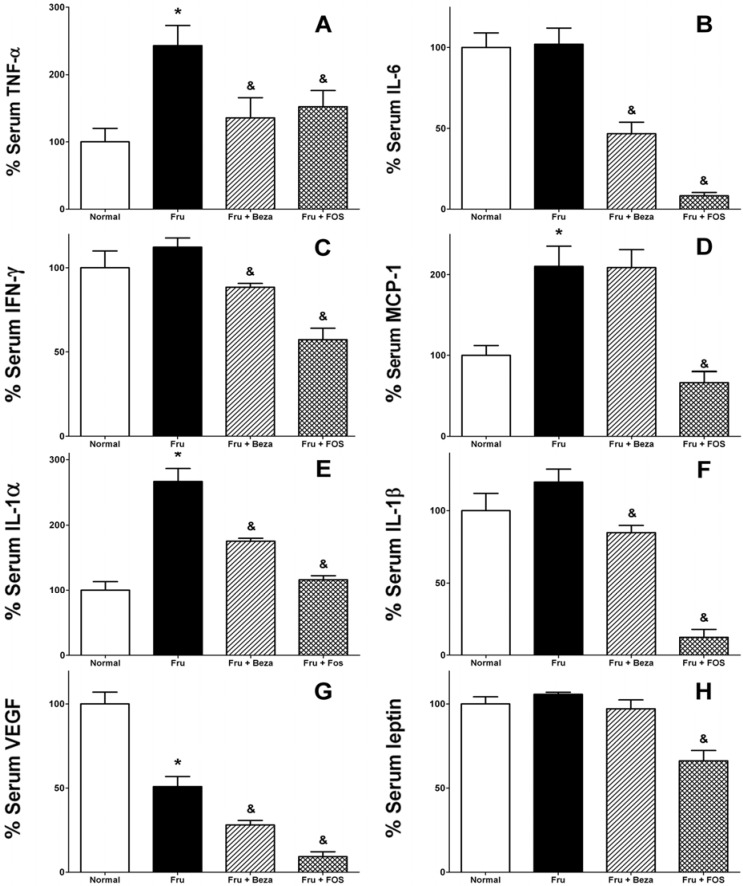
Effect of daily administration of FOS fraction from *P. decompositum* on inflammatory cytokines in male Wistar rats with 24 weeks fructose feeding (**A**) TNF-α; (**B**) IL-6; (**C**) IFN-γ; (**D**) MCP-1; (**E**) IL-1α; (**F**) IL-1β; (G) VEGF; (**H**) leptin, Mean ± S.E.M. (n = 6). * Statistically significant compared with the normal group; statistically significant compared to the group treated with Fru (*p* < 0.05). Normal: normal rats without treatment; FRU: rats treated with high fructose; Fru + Beza: obese rats treated with bezafibrate; Fru + FOS: obese rats treated with FOS.

Figure 1 shows the effect of FOS fraction on the serum levels of eight different obesity-related cytokines, which are closely associated to the inflammatory process. There was an increase of TNF-α in animals with a rich-fructose diet with respect to the normal group (242.65%, Figure 1A). On the other hand, treatment with bezafibrate and FOS fraction showed a decrease (55.95% and 62.78%, respectively) of this cytokine compared to the group with rich-fructose diet, and these values were close to those observed in the normal group (Figure 1A). No difference was observed in IL-6 and IFN-γ between rich-fructose diet and normal control groups, but those treated with FOS fraction (8.08% and 50.99%, Figure 1B and Figure 1C, respectively) and bezafibrate (45.75% and 78.76%, Figure 1B and Figure 1C, respectively) showed a clear decrease in these cytokines. MCP-1 showed an increase in fructose-treated animals compared to the normal group (210.22%, Figure 1D), and for this cytokine, only FOS fraction reduced notoriously blood levels of this marker (31.64%) compared to the Fru group. According to Figure 1E, IL-1α was considerably increased in the rich-fructose diet-treated group (266.48%) with respect to the normal group; for groups that received bezafibrate and FOS fraction, the IL-1α level was not as high as that observed in the Fru group (65.79% and 43.53% less respectively). IL-1β showed an increase in animals that received fructose compared to normal animals, while a decrease of this cytokine was produced in animals treated with bezafibrate (70.93%). However, a bigger effect was observed with FOS fraction (10.36%, Figure 1F).

An interesting effect was observed in the VEGF cytokine. Levels of VEGF were decreased in Fru, Fru + Beza and Fru + FOS groups, compared with the normal group (50.88%). Groups treated with bezafibrate and FOS fraction showed a greater effect than the group treated with only fructose (55.14% and 18.08%, Figure 1G). Finally, leptin levels were not affected by fructose alone or bezafibrate. However, FOS caused a reduction (*p <* 0.05) of this hormone compared with fructose-treated control group (62.59%, Figure 1H).

## 4. Discussion

The water extract of *P*. *decompositum* was chosen due to the use thereof as treatment of folk medicine in Mexico. From the aqueous layer compounds, a carbohydrate type compound has been isolated and reported as the principal compound responsible for the hypoglycemic activity [4]. Once the FOS fraction was obtained and characterized, this material was evaluated in male Wistar rats to evaluate the potential of FOS fraction on process inflammatory and dyslipidemia effect observed in rats with fructose-induced obesity. Such a study is expected to provide more information in the field of the use of this plant in the anti-inflammatory and hypolipidemic activities.

FOS fraction administration did not affect glucose levels in any of the treated groups in glycaemia test. Additionally, there was no difference in groups treated with bezafibrate and FOS fraction for 12 and 24 weeks with respect to tolerance test glucose. For the isotonic saline solution-treated group there was a significant difference compared to the normal group at 120 min. On the other hand, bezafibrate and FOS fraction groups showed no difference at 120 min, compared with the group Fru. In other studies, fructose has been mainly used around 60% on food, which leads to metabolic disorders in less time, developing intolerance to the glucose and hyperinsulinemia. In this study, we used a lower dose than that reported for the hypoglycemic effect.

Food consumption indicates caloric intake, which undoubtedly is directly related to weight gain. Although these measurements were made at the beginning of the experiment, the conditions throughout the experiment in which animals were kept (4 rats/box), did not allow to determine the suitable amount (g) of food consumed per day per animal. However, this aspect should be considered in further studies with the FOS fraction to determine the impact of food intake in weight gain.

Recent studies confirm changes in plasma lipid profile, with no apparent weight gain after 10 weeks of intermittent access to fructose solution 12.5% [21]. Body fat gain can lead to chronic changes in leptin and insulin sensitivity, through the loss of hormonal satiety signals [22,23,24]. Other adverse effects developed by the increased intake of fructose include negative effects on cardiovascular and renal functions [25,26,27]. Finally, high fructose intake is in producing signs of metabolic syndrome in adult Wistar rats [28]. The accumulation of fat and high triglyceride levels observed in rats fed with a rich-fructose diet in the present study explains the increase in body weight. FOS fraction, as other dietary fiber mechanisms on metabolic health, is speculated to be a result of changes in intestinal viscosity, nutrient absorption, rate of passage, production of short chain fatty acids and production of gut hormones [29].

We examined the inflammatory process in response to the excess of fat tissue in HFCS-treated rats. The inflammatory profile of the HFCS-treated rats exhibited by pro-inflammatory cytokines, suggests that this rat model is accessible and shares some similarities with the metabolic syndrome in human beings, such as obesity, impaired glucose tolerance, hyperinsulinaemia and insulin resistance. Several reports have shown increased TNF-α, IL-6, resistin and leptin expression in obesity, type 2 diabetes, cardiovascular disease and metabolic syndrome [30,31].

Among numerous cytokines, TNF-α was identified as the most important pro-inflammatory mediator in metabolic inflammation, as this cytokine is overexpressed in the adipose tissue of rodent models of obesity [32]. Therefore, the overproduction of TNF-α is an important feature of obesity and metabolic inflammation, contributing significantly to insulin resistance. Another pro-inflammatory cytokine, IL-6, is considered to cause insulin resistance in skeletal muscles and liver due to defects in phosphorylation of insulin receptor substrate, causing a decrease in gluconeogenesis and an increase in glycogenolysis [33,34,35]. Therefore, the reduction of TNF-α and IL-6 (two pro-inflammatory cytokines) by FOS fraction treatment can be interpreted as a consequence of its beneficial anti-inflammatory effect.

IFN-γ is also considered a potent pro-inflammatory cytokine that is secreted by activated lymphocytes and is capable of inhibiting the peroxisome proliferator activated receptor gamma (PPAR-γ), an important transcription nuclear factor of anti-inflammatory cytokines [36,37]. The FOS fraction could be capable of increasing the expression of PPAR-γ and decrease IFN-γ; this would explain the reduction of pro-inflammatory cytokines. Activation of these pro-inflammatory cytokines (TNF-α, IL-1, IL-6, IL-8, IL-18 and IFN-γ) generates a chronic inflammation due to a shift in the balance between production of pro-inflammatory cytokines and the production of anti-inflammatory cytokines, such as IL-10 and adiponectin [38,39].

In this research, the HFCS-treated rats displayed large increased TNF-α, MCP-1 and IL-1α serum levels. On the other hand, serum protein levels of VEGF showed a decrease. All other cytokines showed similar values to the normal group. The HFCS-treated rats have higher TNF-α, observed in obese and type 2 diabetes patients [40]. Also, a direct relationship between elevation of TNF-α and hyperinsulinemia has been reported [41]. IL-6% protein levels were slightly higher in HFCS rats than in normal rats. In HFCS rats, the contribution of fat tissues to serum IL-6 levels is low, as IL-6 is also secreted from numerous other cell types, including immune cells, fibroblasts, endothelial cells and striated muscle cells [42]. Adipocytes contribute with 10%–30% of total circulating IL-6 and other cells produce the rest. Chronic inflammation in fat tissues encompasses macrophage infiltration, which produces several adipokines that contribute to the insulin resistance in obese animals [43].

Leptin receptors belong to the cytokine class I receptor family, and several published works have reported that there is an increased inflammatory response associated with the presence of hyperleptinemia without obesity and that leptin is able to control TNF-α production and activation by macrophages [44,45]. Thus, the low leptin levels observed in this work explain the anti-inflammatory effect caused by FOS fraction in the rich-fructose-fed rats.

Several evidences have shown that chronic activation of intracellular pro-inflammatory pathways within insulin target cells can lead to obesity-related insulin resistance. At the molecular level, the mechanisms by which resistance is generated can be multiple and vary from one individual to another. The most common are free fatty acids and their metabolites, TNF-α, IL-6, IL-1β, macrophage migration inhibitory factor (MIF) and hormones secreted by adipose tissue such as resistin [34,43].

Although it would be quite valuable to know if FOS fraction affects insulin levels in order to understand the possible mechanism of hypoglycemic action, in this study, the response to insulin by FOS fraction did not measure. Therefore, the measurement of this hormone in new studies with FOS fraction should be considered.

FOS induces profound metabolic changes by modulating the composition and the activity of the intestinal microbiota, generating anti-inflammatory mechanisms, and immunomodulatory effects. These changes may be the result of reduced hepatic exposure to intestinal products that promote the release of TNF-α from adipose and hepatic tissue macrophages [11].

Different issues contribute to obesity and the pathogenesis of its metabolic disorders, such as genetic, inflammatory, and environmental factors. Among them, diet is the most important one, even though its role in obesity is more complex than a simple fat accumulation. In this regard, FOS could be directly involved in the inhibition of the monoglycerides or nonesterified fatty acids uptake in the small intestine, or modifying gastrointestinal functions related to fat absorption [10].

Measurements of HDL and LDL would be helpful to evaluate hypocholesterolemic effect, mainly to establish whether the decrease observed in total cholesterol observed in the group treated with FOS fraction correlated with increased HDL and decreased LDL. However, our study is preliminary to determine the potential effect on dyslipidemia. In general, concentration of total cholesterol is an important factor for expansion by chronic diseases such as obesity and hypertension, among other risks; HDL and LDL values should nonetheless be determined in further studies.

To clarify the mechanism of the effects of FOS on obesity, our future studies will be focused on hepatic lipid metabolism-related gene expression and quantification of anti-inflammatory cytokines such as adiponectin and IL-1, IL-2, IL-4, IL-10, IL-13 mainly.

## 5. Conclusions

According to our results, FOS fraction obtained from the root of *P*. *decompositum* produces anti-inflammatory effect and an inhibitory effect on the increase in weight, production of triglycerides and cholesterol in obesity fructose-induced in Wistar rats. This could be explained by the fact that FOS improves intestinal digestion, prevents accumulation of fat, and avoids the increase of inflammatory cytokines linked to obesity. The FOS fraction may be a valuable therapeutic agent in preventing insulin resistance, as well as the development and progression of obesity and metabolic syndrome complications.
